# The Missouri Valley Dental Society

**Published:** 1870-03

**Authors:** 


### THE MISSOURI VALLEY DENTAL SOCIETY.

The Missouri Valley Dental Society met in the office of Dr. Thomas C. Chadduck, Nebraska City, Neb., Tuesday and Wednesday, Jan. 11th and 12th, 1870, the President, Dr. E. J. Woodbury, in the Chair.

The minutes being read and approved, the President opened the regular business of the session in an address on the treatment of exposed nerves, which was full of practical information.

Dr. Sanborn addressed the Society on extracting teeth for patients of hemorrhagic diathesis, giving the treatment before as well as subsequent to the operation.

Dr. Thomas, of Nebraska City, gave an address on treatment of irregularities of teeth. These, as well as other subjects, were thoroughly discussed.

There was an essay read on "Vital Phenomena," which contained some ideas so out of the ordinary channel of thought that it called out considerable discussion.

The second day was spent in clinic; mallet contour fillings being the order of the day, which, it is with pleasure we inform our Eastern professional brethren, would compare favorably with such of their contour fillings as we have had the pleasure of seeing. The mallet has not entirely superceded hand pressure, in all cases, yet it must be very flattering to Prof. Atkinson to see the improvement that has been made in filling teeth since its introduction. Harmony and a free interchange of ideas characterize all our meetings, thus far.

